# The American Medical Association

**Published:** 1882-07

**Authors:** 


### ARTICLE IX.

### *The American Medical Association.*

The thirty-third annual session of this representative Association met at St. Paul, Minnesota, on June 6th, at 11 A. M. In the absence of Dr. Woodward, the President, who is now in Europe on account of his health, Dr. Hooper, Vice President, delivered the usual address. The Governor of Minnesota also made an address, welcoming the delegates to his State.

Protests were read from a number of Societies, against admitting the delegates from the New York State Medical Society. Letters were also read from Prof. S. D. Gross,

Dr. Woodward and Dr. Lewis Sayre, the latter to the New York State Medical Society, refusing to act as their delegate, on account of his not agreeing with their action regarding the Code of Ethics.

On the examination of the register there were found to be over 950 members present.

On June 7th, after the reading of several invitations and announcing the nominations, Dr. Dennison, of Colorado, offered a resolution, with the view of correcting a misconduct which exists in the public mind, and to some extent among members of the medical profession, as to the liberty of action authorized by the Association in the treatment of disease.

The committee appointed at the last meeting to consider the advisability of journalizing the Transactions, reported favoraly, and action was taken looking forward to establishing a journal.

J. A. Octerlony, M. D., then read an address in Medicine, after which the Judicial Council made their report, in which they unanimously decided that the New York State Medical Society having fallen below the standard required by the Association, in adopting certain resolutions antagonistic to the Code of Ethics of the Association, were not entitled to representation. This was the only report that could be expected in the case, and though the men who it affected have, through the organ, attempted to disparage the Association in its usefulness, it will undoubtedly give a severe check to those medical men who wish to trade in their profession. Before adjourning Dr. Marcy, of Boston, delivered an address on Obstetrics.

June 8th was, for the best part, consumed by routine business, appointment of committees, etc. During the latter part of the day, Dr. Byrd, of Illinois, read an address on Surgery, and Dr. Gihon, of the United States Navy, delivered the address on State Medicine.

June 9th, session opened at 10 o'clock by the reports of the different officers. Dr. Keller offered a resolution in favor of cremation; referred to the Committee on State Medicine. Dr Dennison's resolution, offered on June 7th, reported above, was laid on the table. The resolution offered by Dr. Gihon, United States Navy, regarding expert testimony, was agreed to.

Cleveland was chosen as the next meeting place, and the following gentlemen were elected officers for the ensuing year: President, J. L. Atlee, of Lancaster; Vice Presidents, E. Grissom, N. C.; H. A. Saul, Ark.; J. A. Octerlony, Ky.; H. S. Orle, Cal.; Treasurer, R. J. Dunglison, Philadelphia; Librarian, C. H. A. Kleinschmidt; Secretary, W. B. Atkinson, Philadelphia; Committee on Publication, Drs. Atkinson, Cohen, Lee, Woodbury, Dunglison and Trike, all of Philadelphia.

After addresses by Dr. Goodwillie, of New York, on Dental and Oral Surgery, and on Diseases of Children, by Dr. S. E. Bury, of Washington, miscellaneous business was transacted, and the Association adjourned. From the importance of the subjects discussed, the meeting can be said to have been most successful.—*Med. Register.*

